# CRISPR/Cas9-induced *shank3b* mutant zebrafish display autism-like behaviors

**DOI:** 10.1186/s13229-018-0204-x

**Published:** 2018-04-02

**Authors:** Chun-xue Liu, Chun-yang Li, Chun-chun Hu, Yi Wang, Jia Lin, Yong-hui Jiang, Qiang Li, Xiu Xu

**Affiliations:** 1 Division of Child Health Care, Children’s Hospital of Fudan University, 399 Wanyuan Road, Shanghai, 201102 China; 2 Center for Translational Medicine, Institute of Pediatrics, Shanghai Key Laboratory of Birth Defect, Children’s Hospital of Fudan University, 399 Wanyuan Road, Shanghai, 201102 China; 3 Department of Pediatrics and Neurobiology, Duke University School of Medicine, Durham, NC 27614 USA

**Keywords:** *shank3*, CRISPR/Cas9, Zebrafish, ASD, Social behavior, Animal model

## Abstract

**Background:**

Human genetic and genomic studies have supported a strong causal role of *SHANK3* deficiency in autism spectrum disorder (ASD). However, the molecular mechanism underlying *SHANK3* deficiency resulting in ASD is not fully understood. Recently, the zebrafish has become an attractive organism to model ASD because of its high efficiency of genetic manipulation and robust behavioral phenotypes. The orthologous gene to human *SHANK3* is duplicated in the zebrafish genome and has two homologs, *shank3a* and *shank3b*. Previous studies have reported *shank3* morphants in zebrafish using the morpholino method. Here, we report the generation and characterization of *shank3b* mutant zebrafish in larval and adult stages using the CRISPR/Cas9 genome editing technique.

**Methods:**

CRISPR/Cas9 was applied to generate a *shank3b* loss-of-function mutation (*shank3b*^*−/−*^) in zebrafish. A series of morphological measurements, behavioral tests, and molecular analyses were performed to systematically characterize the behavioral and molecular changes in *shank3b* mutant zebrafish.

**Results:**

*shank3b*^−/−^ zebrafish exhibited abnormal morphology in early development. They showed reduced locomotor activity both as larvae and adults, reduced social interaction and time spent near conspecifics, and significant repetitive swimming behaviors. Additionally, the levels of both postsynaptic homer1 and presynaptic synaptophysin were significantly reduced in the adult brain of *shank3b-*deficient zebrafish.

**Conclusions:**

We generated the first inheritable *shank3b* mutant zebrafish model using CRISPR/Cas9 gene editing approach. *shank3b*^−/−^ zebrafish displayed robust autism-like behaviors and altered levels of the synaptic proteins homer1 and synaptophysin. The versatility of zebrafish as a model for studying neurodevelopment and conducting drug screening will likely have a significant contribution to future studies of human *SHANK3* function and ASD.

**Electronic supplementary material:**

The online version of this article (10.1186/s13229-018-0204-x) contains supplementary material, which is available to authorized users.

## Background

SHANK3 is a master scaffolding protein enriched at the postsynaptic density of excitatory glutamatergic synapses in the brain that has critical roles in synaptogenesis and synaptic function [1–6]. *SHANK3* is the key gene implicated in the neurobehavioral features of individuals with chromosome 22q13.3 deletion syndrome or Phelan-McDermid syndrome (PMS) [7, 8]. Moreover, genetic studies have identified point mutations in the *SHANK3* gene in cases of autism spectrum disorder (ASD) that establish the causal role of *SHANK3* mutations in ~ 1% of individuals with ASD [9–11].

Animal models of ASD that mimic *SHANK3* genetic detects have facilitated a better understanding of the underlying molecular mechanisms and development of more effective treatments [2, 12]. More than a dozen different lines of *Shank3* mutant mice have been generated and characterized [4, 13–15]. Almost all *Shank3* mutant mice exhibit some of the core behavioral features of ASD [4, 13, 14]. Despite significant advantages, there are clear disadvantages associated with the use of rodent models. For example, it remains difficult to scale up for high-throughput drug screening in rodent models [12]. Compared to rodent models, zebrafish (*Danio rerio*) exhibit much more efficient reproduction, rapid external development [12, 16, 17], and optical transparency [17]. Previous studies have shown that the gene orthologous to human *SHANK3* is duplicated in zebrafish as *shank3a* (in chromosome 18) and *shank3b* (in chromosome 4) [18, 19]. Transient knockdown of both *shank3a* and *shank3b* expressions by morpholino method has been reported [19, 20]. However, previously, the analysis of developmental and behavioral characteristics was only conducted within 5 days of post-fertilization (dpf), an early stage of development [19]. In the present study, we generated and characterized the first CRISPR/Cas9 engineered *shank3b* loss-of-function mutation that is stably transmitted in zebrafish. This model will enable a comprehensive study of a mechanistic link between *shank3* loss-of-function and ASD and provide a new experimental platform for high throughput drug screening in the future.

## Methods

### Generation of *shank3b* mutant zebrafish

The detailed procedure for CRISPR/Cas9 editing in zebrafish was described previously [21, 22]. The *shank3b* target in this study was 5′-GGGCGTGTTGTTGCCACGGCCGG-3′ (Additional file 1: Table S1). Injection mixtures included 500 pg of Cas9 mRNA and 120 pg of gRNA. Eighty zebrafish were screened to identify a founder, and the germline mutation frequency was approximately 35%. Mutant sites were verified by comparison to the WT unaffected sequences (chimerism). Chimeric zebrafish were mated onto a Tu background for three generations to obtain *shank3b*^*+/−*^ zebrafish. We crossed *shank3b*^*+/−*^ males and *shank3b*^*+/−*^ females to obtain *shank3b*^*+/+*^, *shank3b*^*+/−*^, and *shank3b*^*−/−*^ littermates for all experiments of phenotypic analyses.

### *Tg* (*HuC*: RFP) transgenic line and zebrafish maintenance

The wild-type (WT) Tu zebrafish strain was acquired from the Institute of Zebrafish, Children’s Hospital of Fudan University. The zebrafish were raised and maintained in a standard laboratory environment (28.5 °C) and a 14 h light/10 h dark cycle according to a standard protocol [17, 23]. The *Tg* (*shank3b*^*+/+*^-*HuC*: RFP^+/−^) transgenic line, kindly provided by Dr. Xu Wang (Fudan University), was made via plasmid injection with tol2 mRNA at single-cell stage followed by screening for germline transmission. The vector was generated by inserting the *HuC* promoter [24] upstream of RFP cDNA followed by polyA sequence in a Tol2 destination vector, using multisite Gateway cloning [25]. In order to collect enough eggs efficiently for the RFP imaging experiments, we crossed *Tg* (*shank3b*^*+/−*^-*HuC*: RFP^+/−^) with *Tg* (*shank3b*^*+/−*^*-HuC*: RFP^+/−^) to obtain *Tg* (*shank3b*^*−/−*^*-HuC*: RFP^+/+^) for the experimental group. We crossed *Tg* (*shank3b*^*+/+*^-*HuC*: RFP^+/−^) and *Tg* (*shank3b*^*+/+*^-*HuC*: RFP^+/−^) to obtain the control group, *Tg* (*shank3b*^*+/+*^-*HuC*: RFP^+/+^).

### RT-qPCR

Real-time quantitative polymerase chain reaction (RT-qPCR) was performed in triplicate, with 4–10 zebrafish per sample. Total RNA was extracted from the larval or adult brains using TRIzol reagent (Ambion, USA). Reverse transcription was performed with a PrimeScript™ RT Reagent Kit (RR037A, TaKaRa, Japan), according to the manufacturer’s protocol. Oligo dT primer (25 pmol) and random 6 mers (50 pmol) were added in 10 μl mixture to efficiently obtain full-length cDNA. RT-qPCR was performed using a LightCycler® 480 apparatus (Roche, Germany) and SuperRealPreMix Plus (Tiangen, China), according to the manufacturers’ instructions. Finally, we used the delta delta CT method to calculate the expression levels. The primers used in this study are described in Table S1 in Additional file 1.

### Larval activity and light/dark tests

A ViewPoint setup combined with an automated computer recording system equipped with VideoTrack software was used to measure locomotor activity. The camera was a Point Grey black-and-white camera with a resolution of 1024 × 768. Videos were recorded for 60 min at 25 fps and were pooled into 1-min time bins. The detection threshold was set to 25. Activity was quantified using Zebralab software. The distance traveled by the larvae in the well was measured to analyze general locomotor activity. For all behavioral analyses, we used a commercial Viewpoint tracking system and custom software written in C++. All behavioral assays were analyzed by experimenters who were blinded to the genotypes. To further analyze the variances of different activity intensity scales among WT, *shank3b*^*+/−*^, and *shank3b*^*−/−*^ zebrafish, we divided the activity equally into five levels (10, 20, 30, 40, and 50) (Additional file 1: Figure S6). Next, we calculated the activity frequency of different activity intensity scales.

Larvae were habituated in 48-well plates, with one animal per well, in our behavioral assessment room, and videos were recorded for 60 min. The diameter of each well was 1.2 cm. After 30 min of habituation, each larva was recorded for a total of 30 min with three light/dark cycles (each consisting of 5 min of light and 5 min of dark). The light intensity for photo motor response (PMR) was 100 lx and the frame rate was 25/s.

### Open-field test

Behavioral experiments were conducted between 10 a.m. and 4 p.m. Each tank was 30 × 30 × 30 cm, with walls made of opaque partitions, and a video camera was suspended above the tank. Adult male zebrafish were allowed to freely swim inside the tank, and videos were recorded for 30 min. The timing of all supplementary videos began at approximately the 10th min.

The thigmotaxis test was performed in the tank divided into two equal zones, a peripheral and a central zone. Adult zebrafish swam freely in the tank. The longer the zebrafish stayed in the peripheral zone, the greater their awareness of danger [12]. The time ratio was the time the zebrafish spent in the peripheral zone divided by the total time spent in tank, and the distance ratio was the distance the zebrafish traveled in the peripheral zone divided by the total distance traveled.

### Shoaling test

Adult male zebrafish were acclimated to the novel tank apparatus for 1–2 min before the test [26]. Videos were recorded for 30 min. The shoaling assessment was performed by measuring the inter-fish distance that represents the average of all distance between each zebrafish in a shoal [27, 28].

### Social preference test

Social preference testing was performed in a standard mating tank (inner dimensions 21 × 10 × 7.5 cm). The tank was separated into two halves by a Plexiglas transparent barrier that allowed the zebrafish to swim freely and was provided sufficient visual information to allow the zebrafish to form a social preference. Behavioral recordings typically started after an acclimation period (1–2 min), when zebrafish usually explored the tank. Videos were recorded for 30 min. The zebrafish behaviors were quantified as a distance distribution or as presence in a zone adjacent to the group or conspecifics. The time ratio was the time spent in the conspecific sector divided by the total time. The distance ratio was the distance traveled in the conspecific sector divided by the total distance traveled. The zebrafish tested were all adult males.

### Kin preference test

The specifications of the mating cylinder were the same as those in the social preference test. Two opaque separators divided the cylinder into three compartments. Videos were recorded for 30 min. Kin preference was represented by the ratio of time spent in the kin sector divided by the total time. The zebrafish tested were all adult males.

### Western blot and antibodies

WT and *shank3b*^*−/−*^ zebrafish brains were prepared for western blotting by dissociating the tissues in lysis buffer (RIPA, Beyotime Biotechnology, China) and 1% protease inhibitor mixture Set I (Calbiochem, San Diego, CA, USA). The lysates were then centrifuged at 12,000 rpm for 5 min, and the supernatant was collected and denatured. 20 μg of total protein were separated on an SDS-PAGE gel (12%) and were blotted onto a polyvinylidene difluoride membrane (Bio-Rad Laboratories, Hercules, CA, USA). Next, the membrane was blocked with 5% bovine serum albumin for 1–2 h at room temperature and was incubated with primary antibodies overnight at 4 °C. The membrane was rinsed and incubated with HRP-conjugated secondary antibodies for 2 h. Finally, chemiluminescent detection was performed with an ECL kit (Rockford, IL, USA). ImageJ software was used for the densitometric analysis (*N* = 3 for each group).

The synaptophysin (1:2000; ab32594) and homer1 (1:1000; ARP40181_P050) antibodies were purchased from Abcam (Cambridge, UK) and Aviva Systems Biology (San Diego, USA), respectively. The β-actin antibody was obtained from Biotech Well (1:2000; code No. WB0196, Shanghai, China).

### Statistical analysis

Statistical analyses were performed using GraphPad Prism software. Simple comparisons between adult *shank3b*^*+/+*^ and *shank3b*^*−/−*^ zebrafish were performed with two-sided unpaired Student’s *t* tests. Analysis of variance (ANOVA) tests were used to compare three genotypes. All the experiments were conducted in triplicate using different samples. *P* values < 0.05 were considered as statistically significant. Values are presented as mean ± SEM.

## Results

### Conservation of human *SHANK* family genes in zebrafish

Previous analyses have suggested that the zebrafish ortholog of human *SHANK3* is duplicated in the zebrafish genome because of the presence of two highly similar copies of human *SHANK3*: *shank3a* and *shank3b* [19]. To further analyze the evolutionary conservation between human and zebrafish, we performed a phylogenetic analysis of the *SHANK* gene family (*SHANK1*, *SHANK2*, and *SHANK3*). As shown in Additional file 1: Table S2 and Figure S1, *SHANK1* and *SHANK2* each have only one homolog that is believed to be an ortholog in the zebrafish genome. Consistent with previous reports [18, 19], we identified two homologs, *shank3a* (1933 aa) and *shank3b* (1643 aa), in the zebrafish genome. *shank3a* and *shank3b* share 59 and 55% identity with human *SHANK3*, respectively (Additional file 1: Table S3 and Figure S2; https://blast.ncbi.nlm.nih.gov/Blast.cgi). *shank3a* displayed an overall 59% identity and 68% similarity with *shank3b* but close to 100% identity in several blocks of amino acids within the protein (Additional file 1: Table S4 and Figure S3). This observation supports that *shank3a* and *shank3b* may have evolved from the same ancestral DNA during their evolution. Although human *SHANK3* was slightly more conserved in *shank3a* than *shank3b*, both of them may be relevant to understand the functions of human SHANK3 protein.

### Generation of *shank3b*^−/−^ zebrafish

**Fig. 1:**
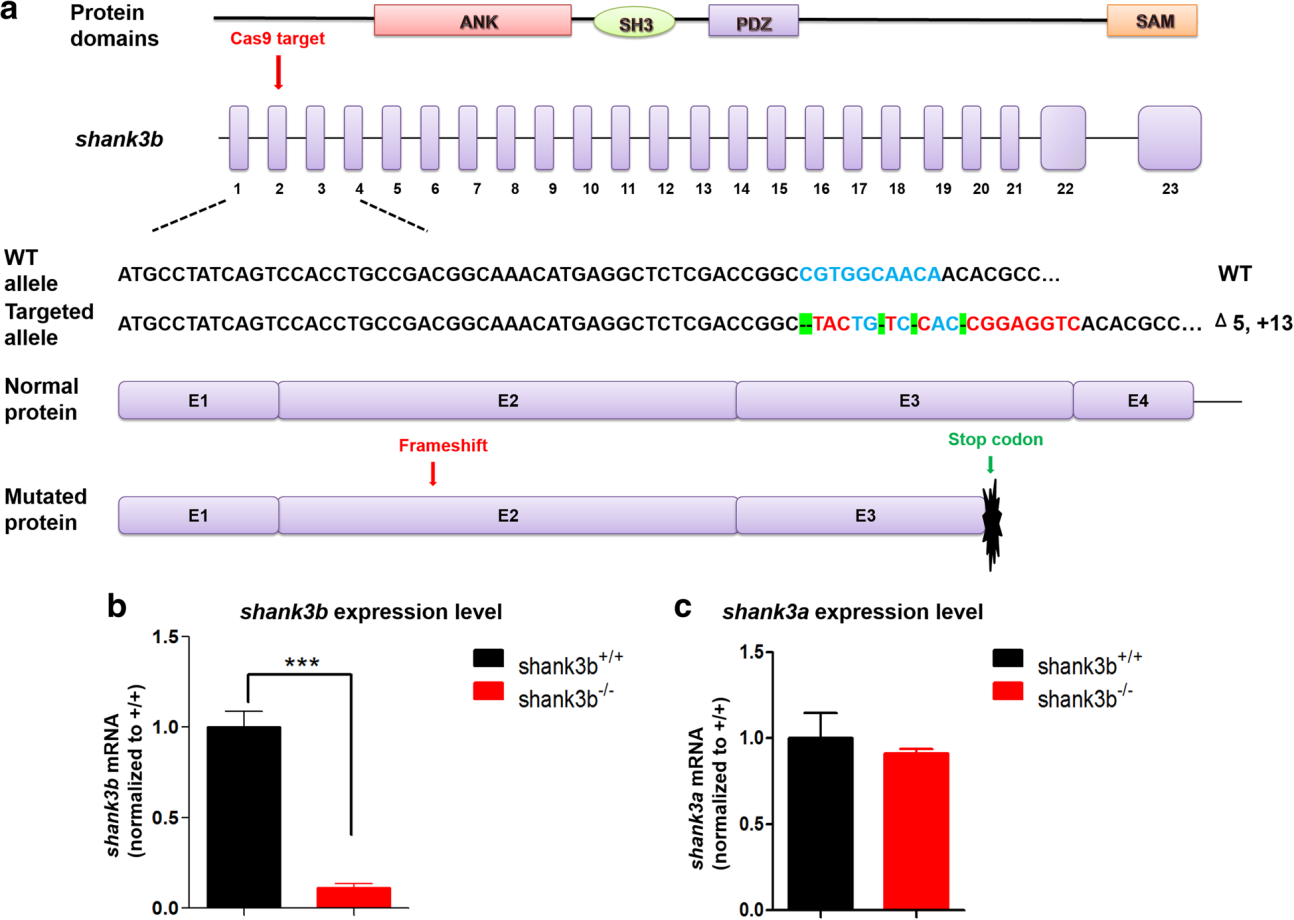
Generation of *shank3b* mutation in zebrafish by CRISPR-Cas9 gene editing. **a** Structure of zebrafish *shank3b* gene and protein. The protein domains (ANK, ankyrin repeat domain; SH3, Src homology 3 domain; PDZ, PSD-95/Discs large/ZO-1 domain; SAM, sterile alpha motif domain) are aligned to the corresponding exons. Exon 2 is the target for CRISPR/Cas9 gene editing in zebrafish *shank3b.* The CRISPR/Cas9-induced mutation (5-base deletion and 13-base insertion) in *shank3b* is shown in annotated *shank3b* mutant sequences. The nucleotides in red are inserted sequences and the green highlighted “-” are deleted nucleotides. **b** Reduced expression of *shank3b* mRNA in the brain of *shank3b*^*+/+*^ and *shank3b*^*−/−*^ adult (6 mpf) male zebrafish analyzed by RT-qPCR. **c** The expression of *shank3a* mRNA in the brain of *shank3b*^*+/+*^ and *shank3b*^*−/−*^ adult (6 mpf) male zebrafish was not affected. Data are shown as mean ± SEM; ***p < 0.001

Zebrafish *shank3b* specific guide-RNA (gRNA) comprising a 23-base sequence was designed for the gene-specific editing of exon 2 of *shank3b*. We generated a *shank3b* mutant by co-injection of Cas9 mRNA and gRNA into zebrafish embryos (one-cell stage). DNA sequencing of target-specific PCR products confirmed that the *shank3b* targeted allele carried a deletion of 5 bases and an insertion of 13 bases, resulting in a frameshift mutation and truncated protein 90 amino acids after the mutation. The mutation disrupted all known functional domains of the shank3b protein (Fig. 1a; Additional file 1: Figure S4). Homozygous mutants for *shank3b* (*shank3b*^*−/−*^) were obtained from the heterozygotes cross (*shank3b*^*+/−*^♂ × *shank3b*^*+/−*^♀) after mating mutants with the original Tu strain for three generations (*shank3b*^*+/−*^). RT-qPCR analysis confirmed that the expression of *Shank3b* mRNA was significantly reduced in *shank3b*^*−/−*^ zebrafish (Fig. 1b), whereas the expression of *shank3a* mRNA was not affected (Fig. 1c). Thus, these results indicated that we have successfully generated a transgenic line of *shank3b-*deficient zebrafish.

### Morphological analysis of *shank3b*^*−/−*^ zebrafish

**Fig. 2:**
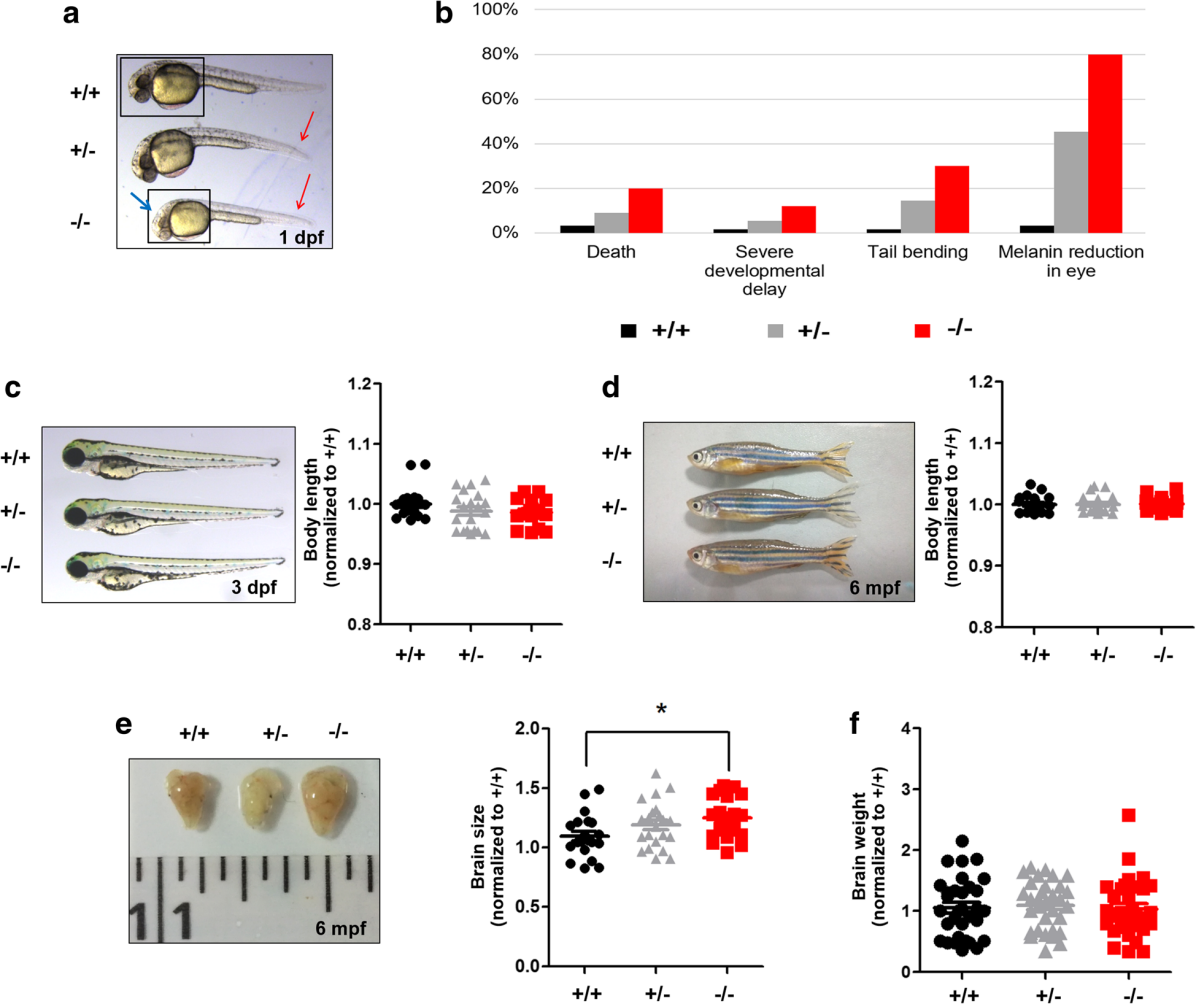
Morphological characteristics of *shank3b*^*−/−*^ larvae and adult zebrafish. **a–b** Abnormal morphological changes in *shank3b*^*−/−*^ and *shank3b*^*+/−*^ larvae at ~ 1 dpf, including severe developmental delay, eye melanin reduction (blue arrow), and tail bending (red arrow) (+/+, *N* = 60; +/−, *N* = 50; −/−, *N* = 50). **c**–**d** Normal morphology and body length of *shank3b*^*+/+*^, *shank3b*^*+/−*^, and *shank3b*^*−/−*^ larvae at 3 dpf (**c**) and adults (6 mpf, male) (**d**) (*N* = 20 for each genotype). **e**–**f** Significantly enlarged brain size (**e**) but normal brain weight (**f**) in adult male *shank3b*^*−/−*^ (6 mpf) compared to WT zebrafish (*N* = 30 for each genotype). *p < 0.05

We measured morphological changes in *shank3b*^*−/−*^ zebrafish to examine the consequences of *shank3b* deficiency during zebrafish development. Compared with *shank3b*^*+/+*^ and *shank3b*^*+/−*^ zebrafish, a significantly greater proportion of *shank3b*^*−/−*^ zebrafish died (*shank3b*^*+/+*^, 3%; *shank3b*^*+/−*^, 9%; *shank3b*^*−/−*^, 20%) and exhibited morphological changes at a very early stage (1 dpf). The morphological changes included neurodevelopmental delay, tail bending, and a reduction of melanin content in eye (Fig. 2a, b). However, over the course of development, these differences in the general phenotypes gradually become less noticeable (Fig. 2c, d). To determine whether there is a maternal or paternal origin effect on the phenotypes observed among *shank3b*^−/−^ zebrafish, *shank3b*^*−/−*^ females were crossed with WT males and *shank3b*^*−/−*^ males were crossed with WT females, respectively. We compared the morphological phenotypes of the offspring from these two breeding schemes and did not find any significant differences (Additional file 1: Figure S5A).

The brain size of adult *shank3b*^*−/−*^ zebrafish was significantly larger than that of *shank3b*^*+/+*^ zebrafish (*p* = 0.01, Fig. 2e), whereas the weight of *shank3b*^*−/−*^ brains was comparable to that of *shank3b*^*+/+*^ and *shank3b*^*+/−*^ brains (Fig. 2f).

### *shank3b*^*−/−*^ larvae exhibited impaired locomotor activity

**Fig. 3:**
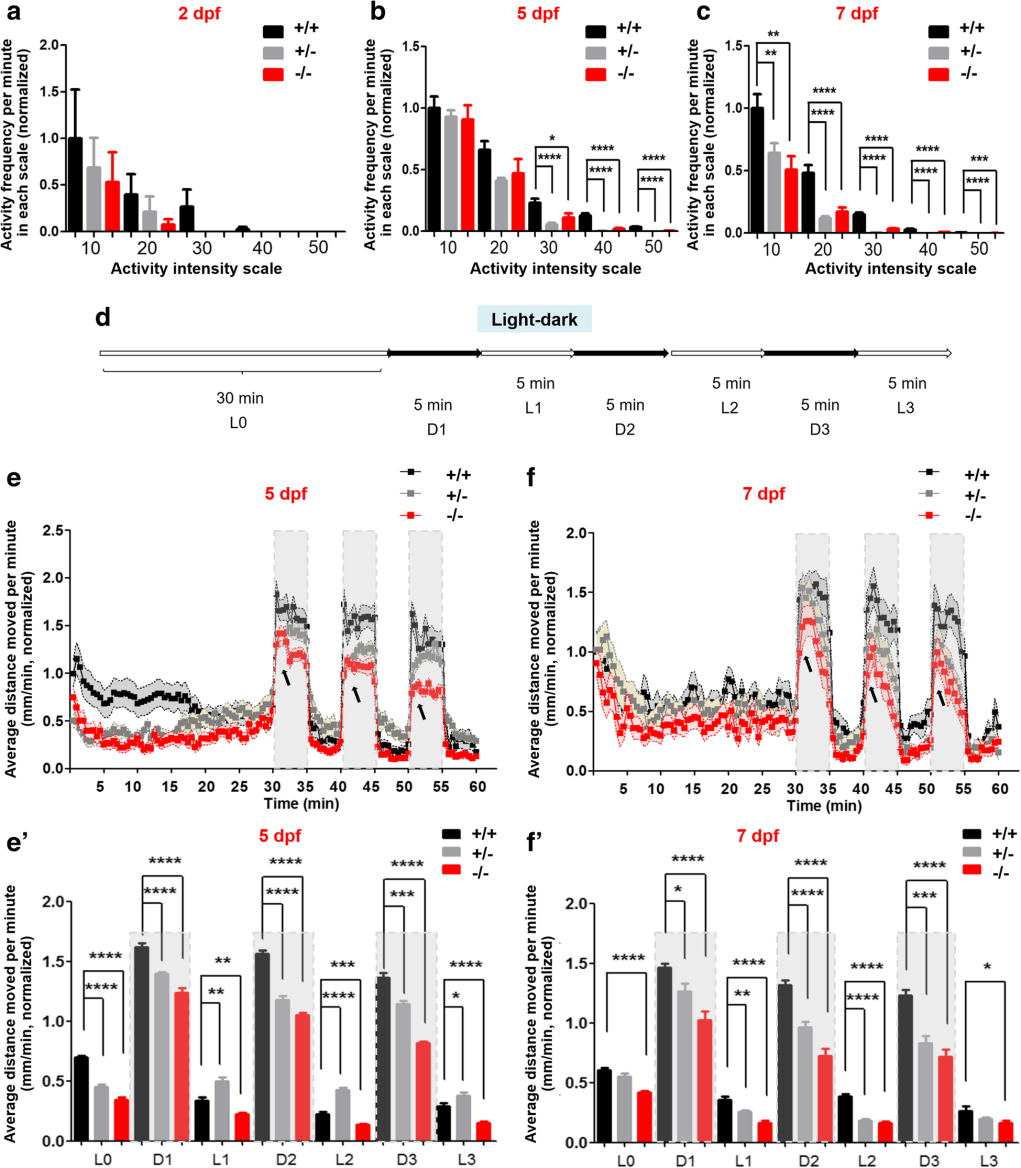
*shank3b*^*−/−*^ larvae displayed impaired locomotion activity. **a–c** Spontaneous activity of *shank3b*^*+/+*^, *shank3b*^*+/−*^, and *shank3b*^*−/−*^ larvae was significantly reduced at 5 and 7 dpf, but not at 2 dpf. The *X* axis shows the intensity scale of the activity and *Y* axis shows the normalized activity frequency traveled by larvae in 1-min bin on each intensity scale (*N* = 24 for each genotype). **d**–**f’** Light/dark test of *shank3b*^*+/+*^, *shank3b*^*+/−*^, and *shank3b*^*−/−*^ larvae at 5 and 7 dpf**.** The activity was recorded during 30 min of light (L0) and three 5-min light/dark intervals (D1/L1, D2/L2, and D3/L3) (**d**). The average distance moved within each 1-min bin under either light or dark conditions is plotted. Experiments were performed at 5 dpf (**e** and **e’**) and 7 dpf (**f** and **f’**). The vertical axis shows the normalized distance (millimeters) traveled by larvae in each 1-min bin. Data are shown as mean ± SEM (*N* = 24 for each genotype); *p < 0.05, **p < 0.01, ***p < 0.001, ****p < 0.0001

To determine whether the loss of function of *shank3b* modulates the larval behaviors during development, the frequency was measured at five activity intensities (10, 20, 30, 40, and 50) among *shank3b*^*+/+*^, *shank3b*^*+/−*^, and *shank3b*^*−/−*^ zebrafish (Additional file 1: Figure S6). The spontaneous activity of individual larva was measured for 30 min in a 48-well plate at 2, 5, and 7 dpf under light exposure (full light strength is 100 lx). Compared with *shank3b*^*+/+*^ larvae, *shank3b*^*−/−*^ and *shank3b*^*+/−*^ larvae exhibited a trend of reduced activity at 2 dpf, but the differences did not reach statistically significance (Fig. 3a). *shank3b*^*−/−*^ and *shank3b*^*+/−*^ larvae moved significantly less than *shank3b*^*+/+*^ larvae at higher activity scales on 5 dpf (Fig. 3b), and at all activity scales on 7 dpf (Fig. 3c).

We also examined the responses evoked by light changes (light/dark switch, 100 lx for brightness and 0 lx for dark). After a 30-min habituation period, each larva displayed relatively stable activity and was recorded for 30 min over three light/dark cycles (each consisting of 5 min in light and 5 min in dark setting per cycle, Fig. 3d). Under continuous illumination, the total distance traveled was measured. Compared with *shank3b*^*+/+*^ larvae, *shank3b*^*−/−*^ and *shank3b*^*+/−*^ larvae traveled significantly less, and *shank3b*^*−/−*^ larvae performed significantly worse than *shank3b*^*+/−*^ larvae. Light-to-dark transitions elicited sudden increases of total distance traveled, while dark-to-light transitions resulted in sudden decreased distance traveled (Fig. 3e, f, e’, f’). However, *shank3b*^*−/−*^ and *shank3b*^*+/−*^ larvae showed fewer responses to changes in illumination (arrows in Fig. 3e, f).

To test whether there is a maternal or paternal origin effect on behavioral phenotypes, we compared larval activity and light/dark switch responses in the offspring of *shank3b*^*−/−*^ female and *shank3b*^*−/−*^ male zebrafish. However, no significant differences were observed among these two groups (Additional file 1: Figs. S5B–5F and S5B’–5F’).

### *shank3b*^*−/−*^ adult zebrafish displayed impaired locomotor activity and abnormal repetitive movements

**Fig. 4:**
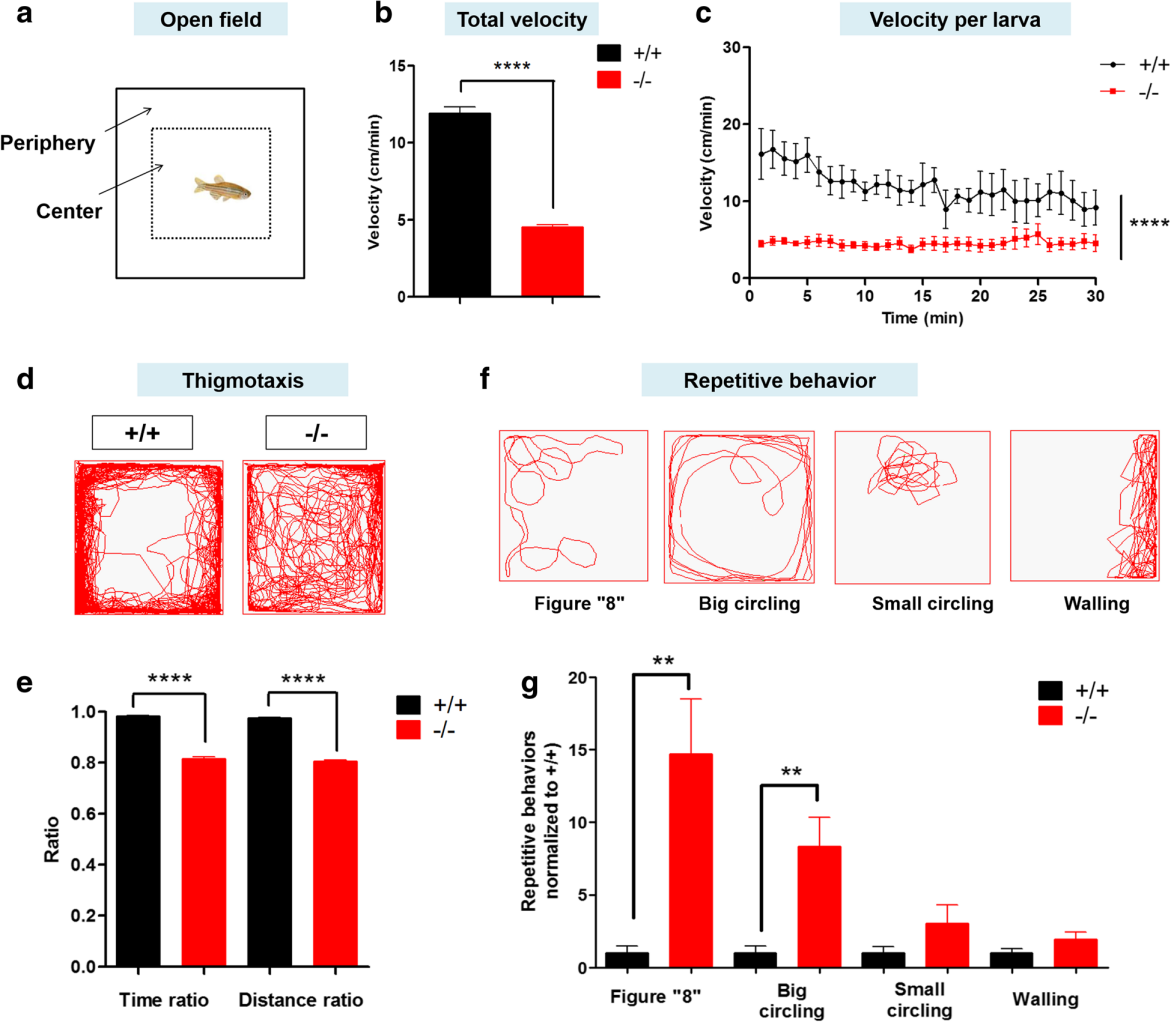
*shank3b*^*−/−*^ adult zebrafish displayed reduced and repetitive locomotion activity in the open-field test. **a** Schematic diagram of the open-field test and thigmotaxis test of adult male zebrafish. In the analysis of thigmotaxis test, the area of the peripheral zone is equal to the center zone (dotted line). **b**–**c** *shank3b*^−/−^ zebrafish at 3.5 mpf showed significantly reduced velocity in the total 60 min period (**b**) and velocity per larva (**c**) in the open field (*N* = 13 for each group). **d** Representative traces of individual *shank3b*^*+/+*^ or *shank3*^*−/−*^ zebrafish in the thigmotaxis test. **e** Ratio for the time spent and distance traveled (periphery divided by the total zone) over 30 min in adult male zebrafish (3.5 mpf). *N* = 13 for each group. **f–g** Representative trace of different types of stereotyped behaviors of *shank3b*^*−/−*^ adult male zebrafish (3.5 mpf). *shank3b*^−/−^ zebrafish had a significantly higher proportion of figure “8” and big circling movements than *shank3b*^*+/+*^. *N* = 13 for each group. Data are shown as mean ± SEM; **p < 0.01, ****p < 0.0001

The locomotor activity of adult *shank3b*^*−/−*^ zebrafish was also examined in an illuminated tank (Fig. 4a). Significantly reduced swimming velocity was observed in *shank3b*^*−/−*^ zebrafish, compared with *shank3b*^*+/+*^ zebrafish (Fig. 4b). Although *shank3b*^*+/+*^ zebrafish displayed reduced velocities with increased time in the tank, *shank3b*^*−/−*^ zebrafish showed steadily lower locomotor activity throughout the examination window (Fig. 4c).

To determine whether disruption of *shank3b* alters thigmotaxis, the two groups of adult zebrafish were assessed for the percentage of time spent and the distance traveled in the center vs. the peripheral zones in a new water tank (Fig. 4a). Compared with *shank3b*^*+/+*^ zebrafish, *shank3b*^*−/−*^ zebrafish spent considerably more time and traveled longer distances in the center of the tank than in the peripheral area (Fig. 4d, e).

When the trajectories of activity and pattern of swimming were analyzed in a blinded fashion, we noticed that *shank3b*^−/*−*^ zebrafish exhibited a significantly higher frequency of stereotypical behaviors (Fig. 4f, g; Additional file 1: Table S5) than *shank3b*^*+/+*^ zebrafish (Additional file 2: Movie S1). The repetitive behaviors include repetitive or stereotypic figure “8” swimming, circling, cornering, and walling (Additional file 3: Movie S2, Additional file 4: Movie S3, Additional file 5: Movie S4, Additional file 6: Movie S5).

### *shank3b*^*−/−*^ zebrafish displayed impaired social preference behaviors

**Fig. 5:**
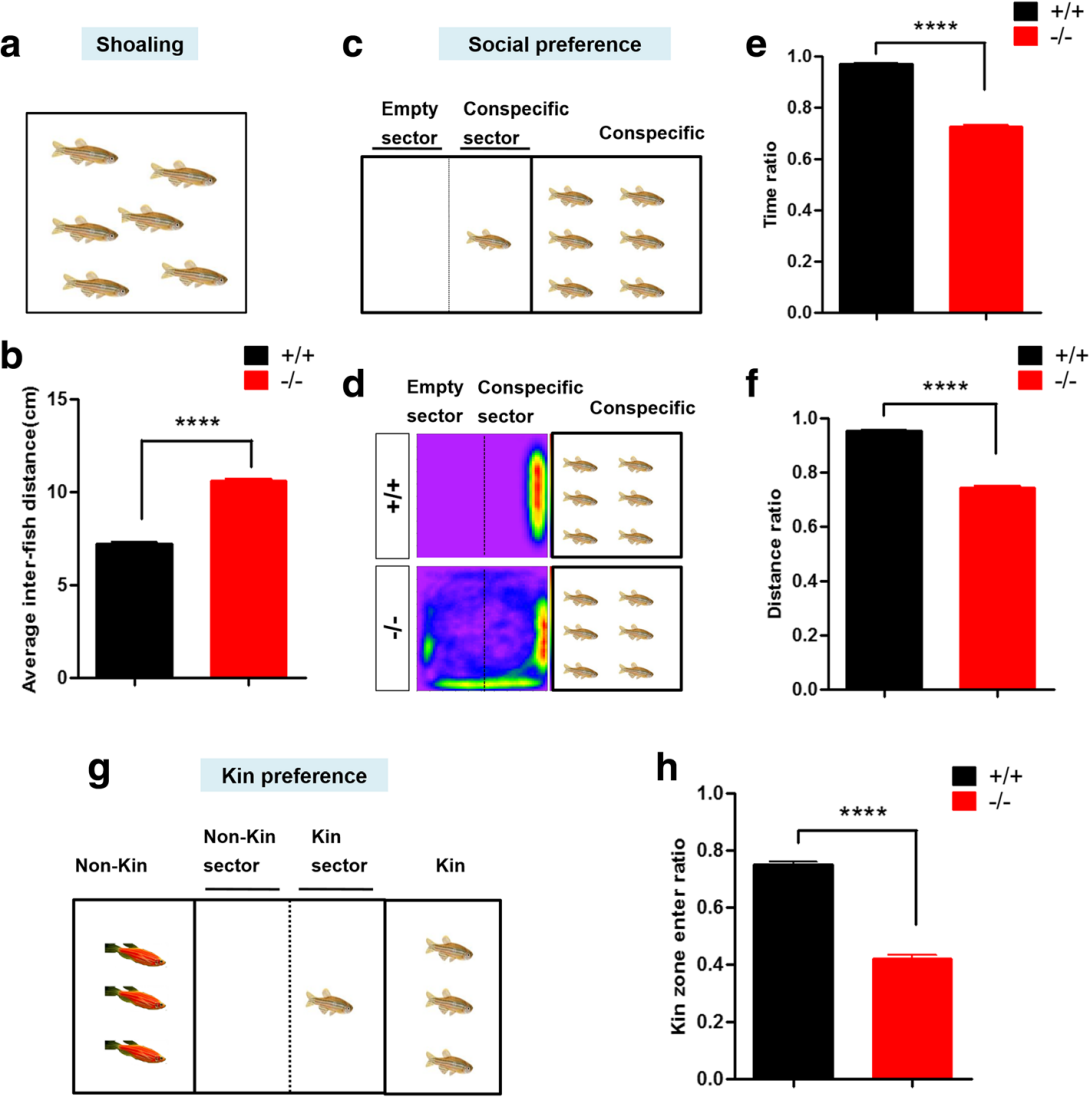
*shank3b*^*−/−*^ zebrafish displayed social interaction defect. **a**–**b** Schematic of shoaling test (**a**) and significantly increased inter-fish distance of adult male *shank3b*^−/−^ zebrafish (3.5 mpf) (**b**). *N* = 18 for each group. **c**–**f** Schematic of social preference test of adult male zebrafish (3.5 mpf) (**c**). Heat map (**d**) shows that *shank3b*^*+/+*^ zebrafish displayed significant higher frequency near a group of zebrafish than *shank3b*^*−/−*^ zebrafish. Time ratio (**e**) and distance ratio (**f**) in the conspecific sector were significantly reduced in *shank3b*^*−/−*^ zebrafish compared to *shank3b*^*+/+*^ zebrafish. *N* = 16 for each group. **g**–**h** Schematic of kin recognition and preference test of adult male zebrafish (3.5 mpf) (**g**) and significantly reduced ratio of kin zone entering in *shank3b*^*−/−*^ zebrafish compared to *shank3*^*+/+*^ zebrafish (**h**). *N* = 10 for each group. Data are presented as mean ± SEM; ****p < 0.0001

It is known that wild-type zebrafish typically swim together in a school that reflects the social nature of the species. We therefore used the shoaling test to assess the social cohesion among homogeneous groups of zebrafish [26, 29]. In this assay, adult *shank3b*^*+/+*^ or *shank3b*^*−/−*^ zebrafish were placed in the testing tank. The average inter-fish distance was measured every 30 s for all pair combinations (Fig. 5a). As shown in Fig. 5b, *shank3b*^*+/+*^ zebrafish typically swim as schools, which is characterized by a short inter-fish distance, a short average diameter of the group, and a clear polarization (Additional file 7: Movie S6), whereas *shank3b*^*−/−*^ zebrafish exhibited larger and looser schools, increased average inter-fish distance, and a greater number of zebrafish swimming away from the group and spending more time outside the group (Additional file 8: Movie S7).

The social preference and interaction tests were subsequently performed using a two-sector tank, divided in the middle with clear Plexiglas to allow visualization. A group of six conspecific zebrafish was placed in the right side, and a single *shank3b*^*+/+*^ or *shank3b*^*−/−*^ test zebrafish was placed on the left side (Fig. 5c). *shank3b*^*+/+*^ zebrafish generally contacted the group on the right side and spent more time in the conspecific sector rather than the empty sector, showing a strong group tendency (Fig. 5d; Additional file 9: Movie S8). In contrast, *shank3b*^*−/−*^ zebrafish spent their time evenly throughout the region and exhibited reduced duration and frequency of social contacts with the peer group (Additional file 10: Movie S9). Quantitatively, compared with *shank3b*^*+/+*^ zebrafish, *shank3b*^*−/−*^ zebrafish exhibited a significantly decreased time ratio (Fig. 5e) and distance ratio (Fig. 5f) in the conspecific sector.

In the related kin recognition and preference test, the zebrafish (*shank3b*^*+/+*^ or *shank3b*^*−/−*^) was placed in the middle of a three-chamber apparatus with Plexiglas dividers, with kin zebrafish placed on the right and non-kin (red color) zebrafish placed on the left (Fig. 5g). *shank3b*^*+/+*^ zebrafish typically spent more time near the kin group (conspecific and same color) than near the non-kin group (Additional file 11: Movie S10), indicating kin recognition and preference. In contrast, *shank3b*^*−/−*^ zebrafish swam in a loose and irregular manner, and the total time spent parallel to conspecifics was much less than that found in *shank3b*^*+/+*^ zebrafish (Fig. 5h; Additional file 12: Movie S11).

### *shank3b* deficiency affected neurodevelopment in larvae

**Fig. 6:**
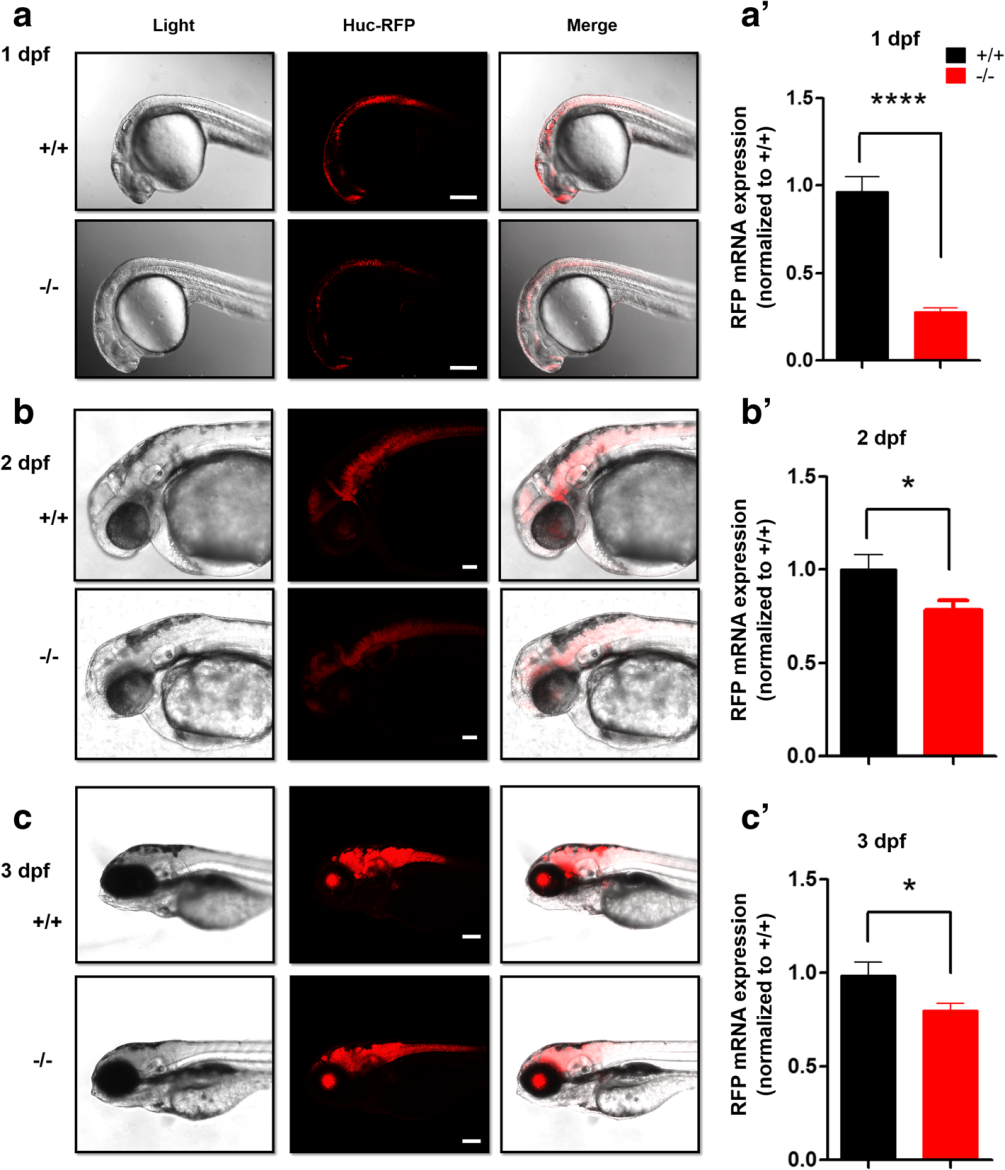
*shank3b* deficiency altered the neurodevelopment in larvae. **a–c** Reduced RFP staining in *shank3b*^*−/−*^ larvae compared to *shank3b*^+/+^ larvae (1 dpf, 2 dpf, and 3 dpf) u*sing Huc:* RFP transgene line zebrafish. The difference is the most prominent at 1 dpf. Scale bar, 100 μm. **a’–c’** RT-qPCR results of RFP expressions from (**a**–**c**) larvae. *N* = 8 for each group. Data are presented as mean ± SEM; *p < 0.05, ****p < 0.0001

To further study neural development, the *HuC-*RFP transgenic line that is widely expressed in the nervous system during embryonic development was used in this study. The *HuC-*RFP transgene, in which the *HuC* promoter drives RFP expression, enables clear and direct visualization of neurodevelopment in transparent larvae (Fig. 6a–c). Compared with *shank3b*^*+/+*^ larvae, the expression of the RFP reporter was significantly reduced in s*hank3b*^*−/−*^ larvae from 1 to 3 dpf, indicating that the neurodevelopment of s*hank3b*^*−/−*^ larvae was altered (Fig. 6a’–c’). In addition, the differences in RFP expression at 1 dpf decreased over time, consistent with the developmental delay shown in Fig. 2.

### *shank3b* deficiency resulted in reduced homer1 and synaptophysin protein levels in the adult zebrafish brain

**Fig. 7:**
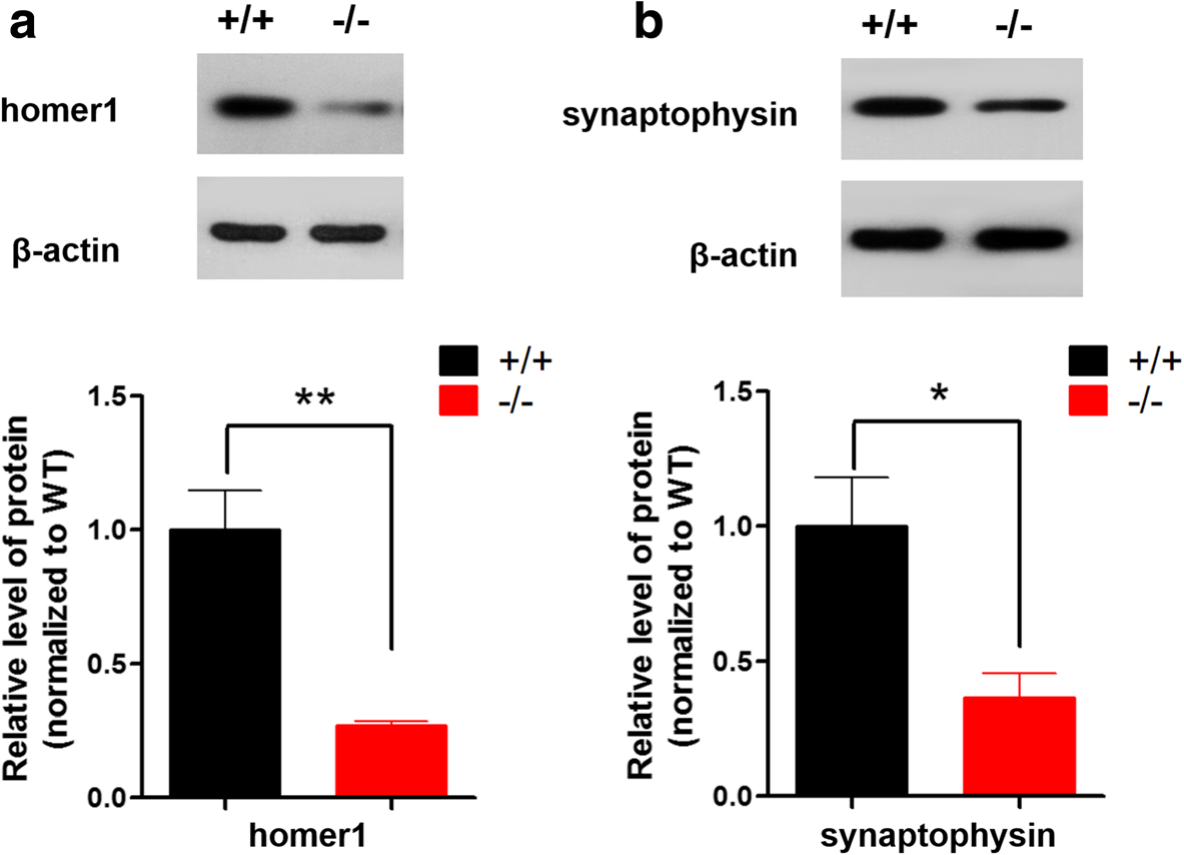
*shank3b* deficiency resulted in the reduction of post- and presynaptic proteins in adult zebrafish brain. **a** Quantitative immunoblot blot analysis showed that the postsynaptic protein homer1 was significantly decreased (27% of *shank3b*^*+/+*^) in the s*hank3b*^*−/−*^ male zebrafish brain relative to *shank3b*^*+/+*^ zebrafish (3.5 mpf, *N* = 3 for each group). **b** The expression of presynaptic synaptophysin protein was markedly reduced in s*hank3b*^*−/−*^ male zebrafish brain compared with that of *shank3b*^*+/+*^ zebrafish (3.5 mpf, 49% of *shank3b*^*+/+*^). *N* = 3 for each group. Data are presented as mean ± SEM; *p < 0.05, **p < 0.01

Shank3 is a core scaffolding protein located at the postsynaptic density [1]. Significantly reduced Homer1, a major postsynaptic protein, is reported in *Shank3* mutant mice [4]. We therefore examined homer1 protein levels in adult s*hank3b*^*−/−*^ zebrafish brains. We found that the level of homer1 protein was significantly decreased (27% of *shank3b*^*+/+*^) in the brain of *shank3b*^*−/−*^ zebrafish (*n* = 3, mean ± SD, 0.27 ± 0.02) compared with *shank3b*^*+/+*^ zebrafish (*n* = 3, 1.00 ± 0.25; Fig. 7a).

*Shank3* deletion has also been reported to impair synaptic transmission, and neurexin and neuroligin mediated trans-synaptic signaling [30]. We investigated whether presynaptic proteins were also affected in the *shank3b*^*−/−*^ zebrafish brain. Synaptophysin is exclusively located in synaptic vesicles and is generally used as a marker for presynaptic terminals [31]. As shown in Fig. 7b, the levels of synaptophysin were markedly decreased in s*hank3b*^*−/−*^ zebrafish (49% of *shank3b*^*+/+*^; *n* = 3, 0.54 ± 0.13) compared with *shank3b*^*+/+*^ zebrafish (*n* = 3, 1.10 ± 0.31).

## Discussion

In this study, we generated the first *shank3b* loss-of-function mutation in zebrafish using the CRISPR/Cas9 gene editing method and reported the morphological, behavioral and neurological characterizations of *shank3b* zebrafish mutants at both early developmental stage and adulthood. The *shank3b* deficiency caused partial lethality during early development as well as defective and delayed neurodevelopment at the larval stage. The brain volume of *shank3b*^−/−^ zebrafish is enlarged but the brain weight is comparable to *shank3b*^+/+^, which may indicate the ventricles in *shank3b*^−/−^ are larger than in WT zebrafish. This observation is reminiscent of the enlarged ventricular size frequently reported in human PMS patients [32, 33]. However, it is interesting to note that the defective and delayed neurodevelopment in *shank3b*^−/−^ larvae becomes less noticeable later in development. The exact reason for the finding is not immediately clear but may support a different functional role of shank3b protein at different developmental stages.

s*hank3b*^−/−^ zebrafish in adulthood display significantly abnormal behaviors while *shank3b*^*+/−*^ zebrafish showed intermediate phenotypes compared to those of *shank3b*^*−/−*^ and *shank3b*^+/+^ zebrafish. The phenotypes observed in *shank3b*^*+/−*^ zebrafish are analogous to the haploinsufficiency of *SHANK3* seen in PMS and *SHANK3*-related disorders [9, 34]. The observed early-stage developmental defects and abnormal behaviors in both *shank3b*^*+/−*^ and *shank3b*^*−/−*^ zebrafish larvae are different from *Shank3* rodent models, in which early developmental defects have not been reported, and phenotypes in heterozygous mutants are generally not significant [4, 35, 36]. The reason for these differences between the two species is not clear. Considering that zebrafish have both *shank3a* and *shank3b* homologs to human *SHANK3*, it is somewhat unexpected or counterintuitive that *shank3b* mutant zebrafish have more prominent phenotypes for survival and behavior. An alternative explanation for the behavioral phenotypes is that the more significant abnormal behaviors in *shank3*^*+/−*^ zebrafish are because behavioral assays in zebrafish are more sensitive than that in rodents.

The ortholog of human *SHANK3* is duplicated in the zebrafish genome as *shank3a* and *shank3b* during teleost evolution [12, 17]. The duplicated and conserved shank3a and shank3b share high identity at the amino acid level and are expected to have a similar function in zebrafish [17]. In a previous study, Kozol et al. reported the knock down of *shank3a* and *shank3b* by morpholino and observed embryonic defects in both morphants and impaired touch-induced startle responses in *shank3a* morphants [19]. However, abnormal ASD-like behaviors were not detected due to the limitations of morpholino technology. It would be interesting to compare the phenotypes of *shank3a* and *shank3b* mutants engineered by CRISPR/Cas9 in parallel or even the phenotypes of *shank3a* and *shank3b* double mutants in the future.

In recent years, the zebrafish has become an attractive alternative model for ASD researchers [19, 27, 37]. Many behavioral assays have been developed in zebrafish models, including the assessment of social interaction, novelty seeking, courtship, inhibitory avoidance, fear and anxiety responses, repetitive/stereotyped behaviors, seizures, and aggression [12, 38–41]. We employed some of the behavioral assays in the analyses of *shank3b* mutant zebrafish and found striking differences in social and repetitive behavioral domains between *shank3b*^−/−^ and *shank3b*^+/+^ zebrafish. For instance, in shoaling and kin-preference assays, *shank3b*^−/−^ zebrafish preferred to swim in loose schools and showed significantly decreased preference for conspecifics. These abnormal behaviors are reminiscent of reduced social interaction in the home cage or abnormal social novelty and preference using the three chamber paradigm reported in several lines of *Shank3* mutant mice [35, 36, 42, 43]. In the open field, *shank3b*^−/−^ zebrafish displayed abnormal locomotor activity, such as figure “8” and “circling” movements that are apparently repetitive. Similarly, repetitive behavior measured by increased self-grooming has been observed in several lines of *Shank3* mutant mice [4, 42]. However, like many other behavioral findings observed in animal models, the challenge remains to determine whether the abnormal behaviors observed in *shank3b*-deficient zebrafish can be directly translated to human *SHANK3*-related ASD. The study of the predictive validity of these abnormal behaviors to ASD may be warranted in the future, when feasible. Positive results could potentially provide further support for the translational value of these behavioral phenotypes. It also remains to be seen if these assays are universally valid and effective for ASD models caused by different genetic defects. Clinical and molecular heterogeneity have been well recognized in ASD in humans [44]. Additional behavioral assays are certainly needed to assess face validity for ASD-like behaviors, and also for common comorbidities such as seizures and cognitive impairments.

Our finding of reduced postsynaptic homer1 protein levels in *shank3b*-deficient zebrafish is consistent with the known function of SHANK3 as a scaffolding protein at the postsynaptic density from studies of *Shank3* mutant mice [4, 45]. This finding, although limited, would suggest that the molecular mechanism-associated SHANK3 deficiency may be conserved between different species. It would be interesting to examine if the same defect occurs in *shank3a-*deficient zebrafish. The finding of significantly reduced synaptophysin protein levels in the brain of *shank3b*^−/−^ zebrafish is novel, as synaptophysin is a known presynaptic protein [31]. This observation implies that *shank3b* deficiency may affect presynaptic function directly or via a trans-synaptic mechanism in zebrafish. Several recent studies have suggested that SHANK3 protein is located at the presynaptic terminus in the brain as well as in dorsal root ganglion neurons in rodents [46]. Our finding in zebrafish also potentially suggests a role of shank3 protein in the presynaptic terminus. Future studies on the presynaptic function of *shank3b*^*−/−*^ are warranted and may shed additional insight in this direction.

The amenability to high-throughput drug screening is a tremendous advantage of the zebrafish model. The list of confirmed ASD-causing genes continues to grow, but the development of targeted molecular treatments significantly lags behind. A validated experimental platform that can translate the genetic discoveries to drug screening at a fast pace is urgently needed. We believe that the *shank3b*^*−/−*^ model described in this study and other similar ASD zebrafish models will lay an important foundation for the development of a productive drug screening program for ASD and may ultimately lead to the discovery of an effective intervention.

## Conclusions

For the first time, we successfully generated a *shank3b*^*−/−*^ zebrafish model that displays robust autism-like behavioral characteristics. Reduced levels of the postsynaptic scaffolding protein homer1 in *shank3b*^*−/−*^ zebrafish suggest a high conservation of the molecular mechanism underlying SHANK3 deficiency among different species. The reduced levels of synaptophysin in the brain of *shank3b*^*−/−*^ zebrafish also provide further evidence supporting the potential role of shank3 in presynaptic terminus. The *shank3b* mutant zebrafish represents a valuable model to dissect the molecular pathogenesis and conduct high-throughput drug screening for *SHANK3*-related disorders in the future.

## Additional files

Additional file 1: **Table S1.** gRNA gene-target sequences, oligonucleotides for PCR knock-out validation, and RT-qPCR probes used in this study. **Table S2.** SHANK family sequences used in this study. **Table S3.** Homology analysis of zebrafish *shank3a* and *shank3b* compared with human *SHANK3*. **Table S4.** Homology comparison between zebrafish *shank3a* and *shank3b*. Table S5. Repetitive behaviors of *shank3b*^*−/−*^ adult male zebrafish (3.5 mpf). **Figure S1.** Phylogenetic tree of evolutionary relationship of SHANK family proteins. **Figure S2.** Homology comparison of zebrafish *shank3a* and *shank3b* with human *SHANK3*. **Figure S3.** Homology comparison between zebrafish *shank3a* and *shank3b*. **Figure S4.** *shank3b* target-mutation in zebrafish via CRISPR-Cas9 system. **Figure S5.** Examination of maternal or paternal origin effects on the morphological and behavioral phenotypes. **Figure S6.** Analysis of activity frequency at different activity intensity scales. (PDF 1211 kb)

Additional file 2: **Movie S1.** WT zebrafish swimming in the open field. (MP4 657 kb)

Additional file 3: **Movie S2.** *shank3b*^*−/−*^ zebrafish swimming in repetitive figure “8” pattern. (MP4 484 kb)

Additional file 4: **Movie S3.** *shank3b*^*−/−*^ zebrafish swimming in repetitive big circling pattern. (MP4 1193 kb)

Additional file 5: **Movie S4.** *shank3b*^*−/−*^ zebrafish swimming in repetitive small circling pattern. (MP4 380 kb)

Additional file 6: **Movie S5.** *shank3b*^*−/−*^ zebrafish swimming in repetitive walling pattern. (MP4 1535 kb)

Additional file 7: **Movie S6.** Performance of WT zebrafish in the shoaling test. (MPG 2510 kb)

Additional file 8: **Movie S7.** Performance of *shank3b*^*−/−*^ zebrafish in the shoaling test. (MPG 2022 kb)

Additional file 9: **Movie S8.** Performance of WT zebrafish in the social preference test. (MPG 2562 kb)

Additional file 10: **Movie S9.** Performance of *shank3b*^*−/−*^ zebrafish in the social preference test. (MPG 1992 kb)

Additional file 11: **Movie S10.** Performance of WT zebrafish in the kin recognition and preference test. (MPG 2894 kb)

Additional file 12: **Movie S11.** Performance of *shank3b*^*−/−*^ zebrafish in the kin recognition and preference test. (MPG 2044 kb)

## Abbreviations

ASD: Autism spectrum disorder
dpf: Days post-fertilization
gRNA: Guide-RNA
KO: Knockout
mpf: Months post-fertilization
PCR: Polymerase chain reaction
RT-qPCR: Real-time quantitative polymerase chain reaction
SHANK3: SH3 and multiple ankyrin (ANK) repeat domain 3
WT: Wild type
