# Supplementary material for: CRISPR/Cas9-induced shank3b mutant zebrafish display autism-like behaviors
Source: Mol Autism. 2018 Apr 2;9:23. doi: 10.1186/s13229-018-0204-x (PMC5879542; doi:10.1186/s13229-018-0204-x)
Supplement: Supplementary file 1 — Table S1. gRNA gene-target sequences, oligonucleotides for PCR knock-out validation, and RT-qPCR probes used in this study. Table S2. SHANK family sequences used in this study. Table S3. Homology analysis of zebrafish shank3a and shank3b compared with human SHANK3. Table S4. Homology comparison between zebrafish shank3a and shank3b. Table S5. Repetitive behaviors of shank3b−/− adult male zebrafish (3.5 mpf). Figure S1. Phylogenetic tree of evolutionary relationship of SHANK family proteins. Figure S2. Homology comparison of zebrafish shank3a and shank3b with human SHANK3. Figure S3. Homology comparison between zebrafish shank3a and shank3b. Figure S4. shank3b target-mutation in zebrafish via CRISPR-Cas9 system. Figure S5. Examination of maternal or paternal origin effects on the morphological and behavioral phenotypes. Figure S6. Analysis of activity frequency at different activity intensity scales. (PDF 1211 kb) [file 13229_2018_204_MOESM1_ESM.pdf]

Additional file 1: Tables

Additional file 1: Table S1. gRNA gene-target sequences, primers for PCR genotyping and RT-qPCR probes used in this study

**Item**	Exon/Intron	Sequence (5'-3')	Amplicon length (bp)	Annealing temp (°C)	Ensemble gene ID
**gRNA target: *shank3b***	ex2	GGGCGTGTTGTTGCCACGGCCGG			ENSDART00000145613
**PCR genotyping**	in1	ATATGGGTCTTGCTTGTATACAGGCGA	500	60	
	in2	GAATCAACAACTCGGTCTGTACCAATCA		60.3	
**RT-qPCR**					
shank3b	ex2	CGGCCGTGGCAACAACAC	245	59	ENSDART00000145613
	ex3	TTAAGCACATCGGTCAGGCTTTGT		59	
β-actin	ex2	CGAGCTGTCTTCCCATCCA	102	60	
	ex3	TCACCAACGTAGCTGTCTTTCTG		60	
shank3a	ex5/6	GGCACTTATTACGCTGCTGGATCTG	191	60	ENSDART00000091805.5
	ex6/7	CATGACGGCAAGCCTGGTGAAT		60.7	
RFP		CATGAAGCTGTACATGGAGGGCAC	219	59.4	
		GACTGCTTAAAGAAGTCGGGGATG		59	

Additional file 1: Table S2. SHANK family gene sequences used in this study

Gene	NCBI reference protein number	Amino acid	Species
SHANK3	NP_277052.1	1731	Human
shank3a	XP_017207498.1	1933	Zebrafish
shank3b	XP_009298459.1	1643	Zebrafish
SHANK1	NP_057232.2	2161	Human
shank1	NP_036441.2	1849	Zebrafish
SHANK2	NP_036441	1849	Human
shank2	XP_021326187.1	1810	Zebrafish

Additional file 1: Table S3. Protein homology analysis of zebrafish shank3a and shank3b and human SHANK3

Gene	NCBI reference mRNA number	NCBI reference protein number	Amino acid	vs. human SHANK3 protein		
				Query cover (%)	Identity (%)	Accession (human SHANK3)
shank3a	XM_017352009.2	XP_017207498.1	1933	95	59	NP_277052.1
shank3b	XM_009300184.3	XP_009298459.1	1643	97	55	NP_277052.1

Additional file 1: Table S4. Protein homology comparison between zebrafish shank3a and shank3b

Gene	NCBI reference mRNA number	NCBI reference protein number	Amino acid	shank3a VS. shank3b	
				Query cover(%)	Identity (%)
shank3a	XM_017352009.2	XP_017207498.1	1933	95	59
shank3b	XM_009300184.3	XP_009298459.1	1643		

Additional file 1: Table S5. Repetitive behaviors of *shank3b*$^{-/-}$ adult male zebrafish (3.5 mpf)

	Figure "8"		Big circling		Small circling		Walling	
*No.	#shank3b$^{+/+}$	shank3b$^{-/-}$	shank3b$^{+/+}$	shank3b$^{-/-}$	shank3b$^{+/+}$	shank3b$^{-/-}$	shank3b$^{+/+}$	shank3b$^{-/-}$
1	0	15	0	2	0	2	4	2
2	2	1	0	8	0	0	3	4
3	0	1	0	6	0	0	0	0
4	0	2	0	6	1	2	2	2
5	0	14	0	5	0	1	4	8
6	0	8	1	1	0	2	2	0
7	1	6	1	0	2	2	2	0
8	0	8	0	2	0	7	0	2
9	1	2	0	4	0	0	0	8
10	0	4	0	0	0	0	3	3
11	2	18	2	2	0	1	0	2
12	0	2	0	0	1	0	0	7
13	0	1	1	3	1	0	0	3

*, Tested adult (3.5 mpf) male zebrafish in this study.

#, Number of each type of repetitive behavior occurring in the examination window.

Additional file 1: Figures

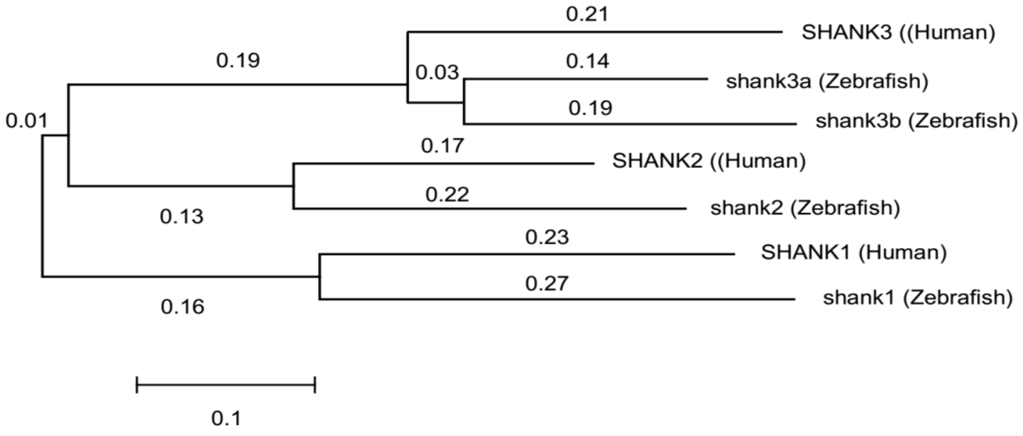

Additional file 1: Figure S1. Phylogenetic tree of evolutionary relationship of SHANK family proteins.

Zebrafish shank3a protein (XP_017207498.1)

Sequences producing significant alignments:

Select: All None Selected:0

Alignments Download GenPept Graphics Distance tree of results Multiple alignment

Description	Max score	Total score	Query cover	E value	Ident	Accession
SH3 and multiple ankyrin repeat domains protein 3 [Homo sapiens]	1725	1725	95%	0.0	59%	NP_277052.1
SH3 and multiple ankyrin repeat domains protein 2 isoform 1 [Homo sapiens]	1336	1336	95%	0.0	46%	NP_036441.2
PREDICTED: SH3 and multiple ankyrin repeat domains protein 2 isoform X2 [Homo sapiens]	1334	1334	95%	0.0	46%	XP_016872878.1
PREDICTED: SH3 and multiple ankyrin repeat domains protein 1 isoform X3 [Homo sapiens]	874	1044	53%	0.0	52%	XP_011525316.1
PREDICTED: SH3 and multiple ankyrin repeat domains protein 1 isoform X1 [Homo sapiens]	870	1039	53%	0.0	51%	XP_011525315.1
SH3 and multiple ankyrin repeat domains protein 1 [Homo sapiens]	868	1037	53%	0.0	52%	NP_057232.2
PREDICTED: SH3 and multiple ankyrin repeat domains protein 2 isoform X3 [Homo sapiens]	755	755	77%	0.0	39%	XP_005277989.1
PREDICTED: SH3 and multiple ankyrin repeat domains protein 2 isoform X4 [Homo sapiens]	609	609	65%	0.0	39%	XP_016872879.1

Zebrafish shank3b protein (XP_009298459.1)

Sequences producing significant alignments:

Select: All None Selected:0

Alignments Download GenPept Graphics Distance tree of results Multiple alignment

Description	Max score	Total score	Query cover	E value	Ident	Accession
SH3 and multiple ankyrin repeat domains protein 3 [Homo sapiens]	1400	1400	97%	0.0	55%	NP_277052.1
SH3 and multiple ankyrin repeat domains protein 2 isoform 1 [Homo sapiens]	964	1103	92%	0.0	54%	NP_036441.2
PREDICTED: SH3 and multiple ankyrin repeat domains protein 2 isoform X2 [Homo sapiens]	963	1102	92%	0.0	54%	XP_016872878.1
PREDICTED: SH3 and multiple ankyrin repeat domains protein 1 isoform X3 [Homo sapiens]	875	1041	54%	0.0	54%	XP_011525316.1
PREDICTED: SH3 and multiple ankyrin repeat domains protein 1 isoform X1 [Homo sapiens]	869	1034	54%	0.0	53%	XP_011525315.1
SH3 and multiple ankyrin repeat domains protein 1 [Homo sapiens]	869	1034	54%	0.0	53%	NP_057232.2
PREDICTED: SH3 and multiple ankyrin repeat domains protein 2 isoform X3 [Homo sapiens]	414	552	62%	8e-120	46%	XP_005277989.1
PREDICTED: SH3 and multiple ankyrin repeat domains protein 2 isoform X4 [Homo sapiens]	287	426	51%	2e-78	47%	XP_016872879.1
SH3 and multiple ankyrin repeat domains protein 2 isoform 2 [Homo sapiens]	237	375	42%	2e-62	54%	NP_573573.2

Additional file 1: Figure S2. Homology comparison of zebrafish shank3a and shank3b with human SHANK3

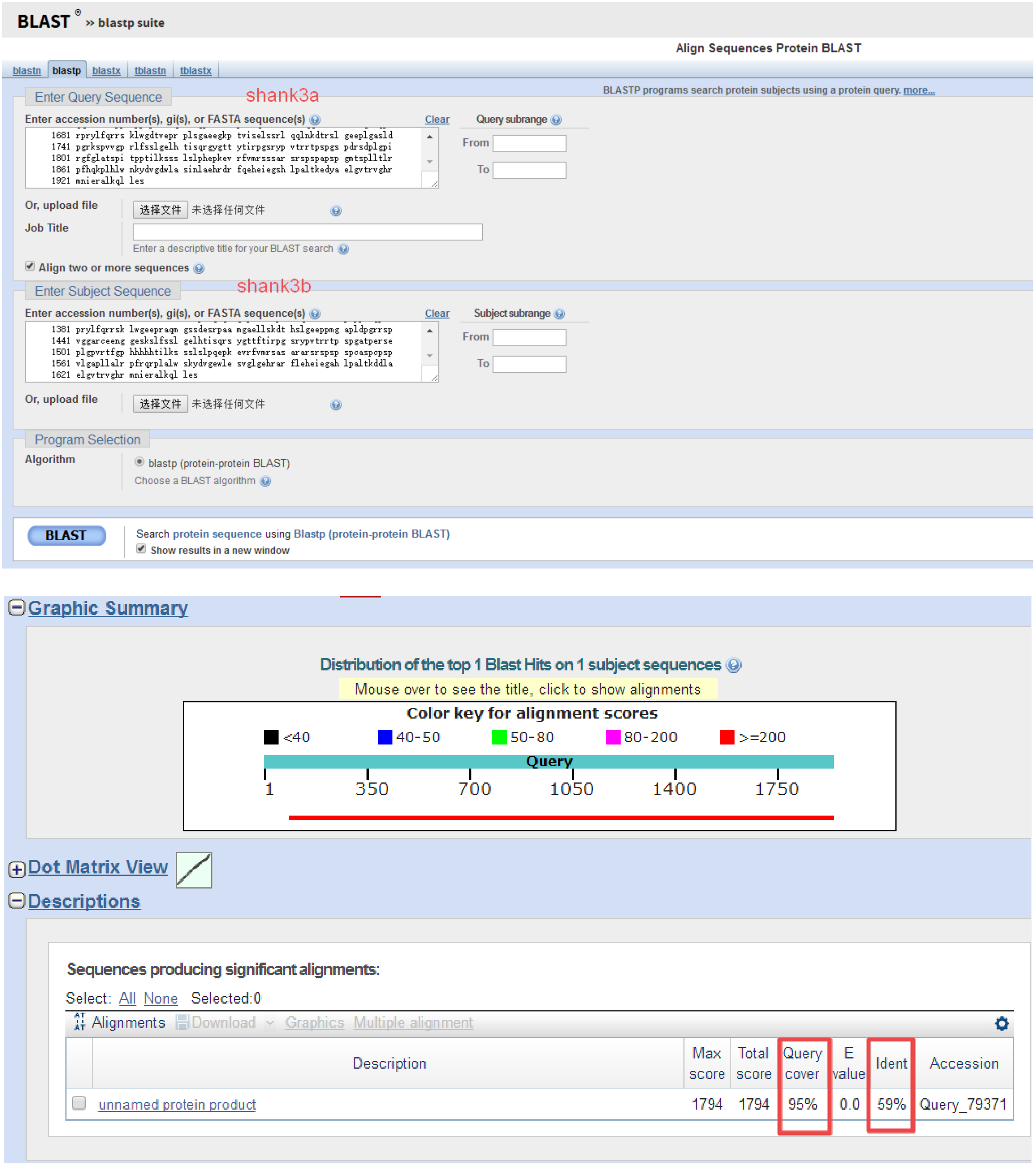

Additional file 1: Figure S3. Homology comparison between zebrafish shank3a and shank3b.

We used the "Protein BLAST" tool of NCBI website (https://blast.ncbi.nlm.nih.gov/Blast.cgi?PAGE=Proteins&PROGRAM=blastp&BLAST_PROGRAMS) to compare the homology of zebrafish shank3a and shank3b.

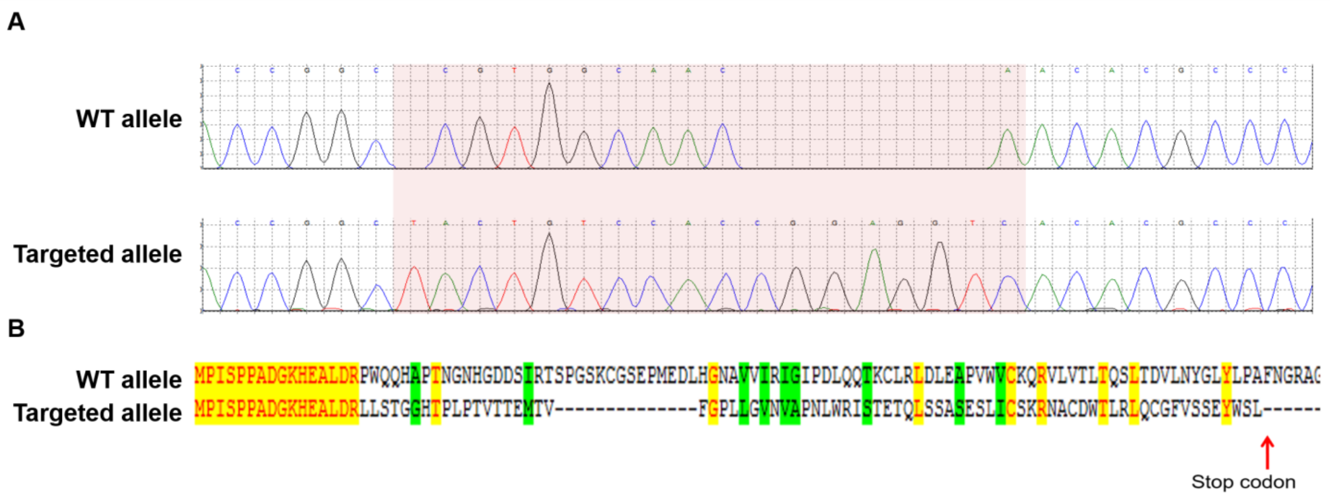

Additional file 1: Figure S4. *shank3b* target-mutation in zebrafish via CRISPR-Cas9 system. (A) Sanger sequencing result of WT and *shank3b* site mutations in zebrafish. *shank3b*$^{-/-}$ zebrafish has a 5-base deletion and a 13-base insertion. **(B)** shank3b protein of WT zebrafish and *shank3b*$^{-/-}$ predicted translated protein sequences are shown. Protein spatial structure prediction results of WT.

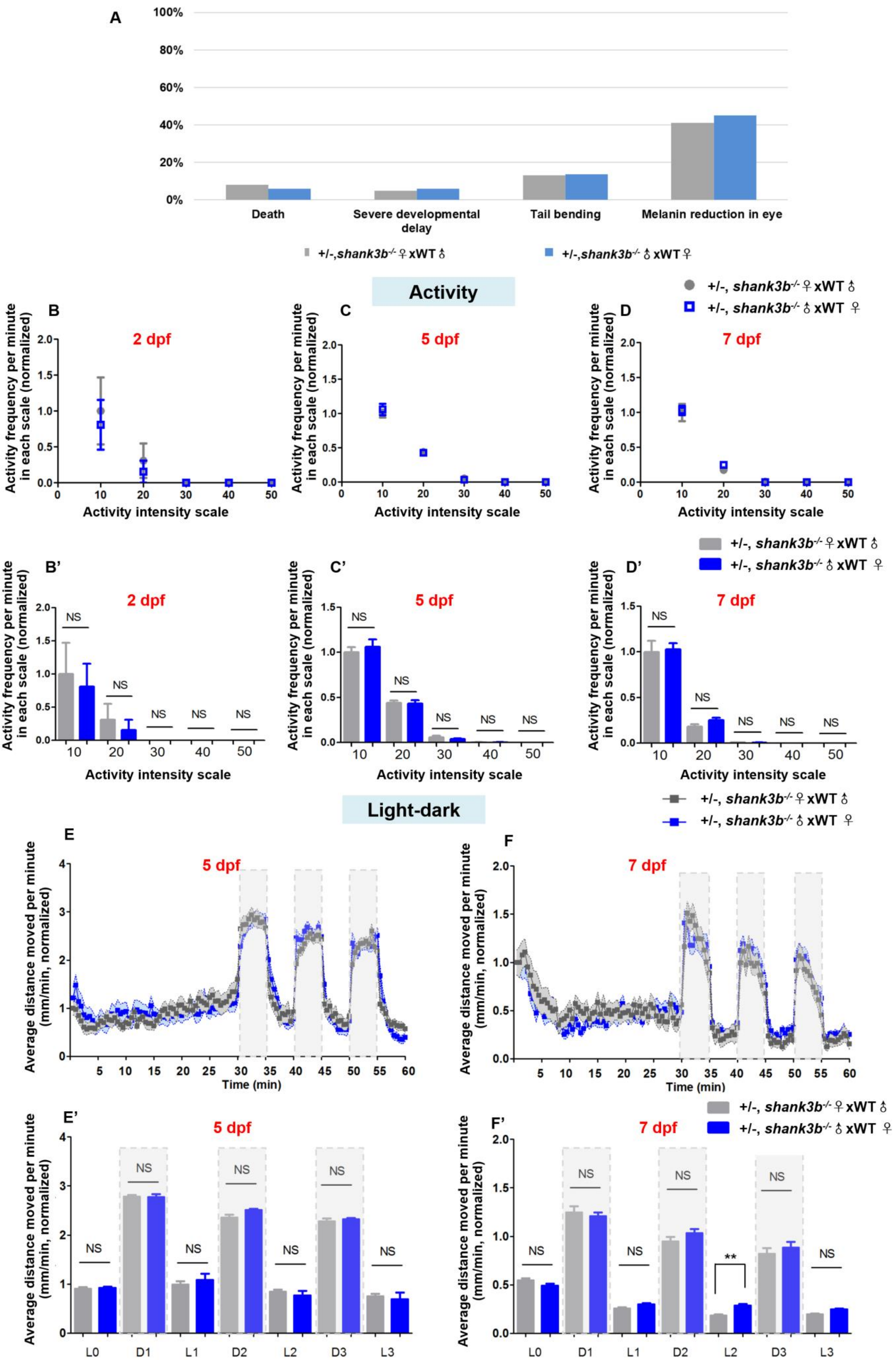
A
Death
Severe developmental delay
Tail bending
Melanin reduction in eye
+/-,shank3b-/- ♀xWT♂
+/-,shank3b-/- ♂xWT♀
Activity
B
2 dpf
C
5 dpf
D
7 dpf
Activity frequency per minute in each scale (normalized)
Activity intensity scale
B'
C'
D'
NS
Light-dark
E
F
Average distance moved per minute (mm/min, normalized)
Time (min)
E'
F'
L0
D1
L1
D2
L2
D3
L3
**

Additional file 1: Figure S5. Examinations of maternal or paternal origin effects on the morphological and behavioral phenotypes. (A) Morphological examination of the *shank3b*$^{-/-}$ ♀ and *shank3b*$^{-/-}$♂ offspring at 1 dpf. **(B-D)** Activity of *shank3b*$^{-/-}$ ♀ and *shank3b*$^{-/-}$♂ offspring at 2 dpf, 5 dpf and 7 dpf. **(E-F)** Light/dark response of *shank3b*$^{-/-}$ ♀ and *shank3b*$^{-/-}$♂ offspring at 5 dpf and 7 dpf. *shank3*$^{+/-}$ ♀ were obtained from crosses of *shank3b*$^{-/-}$ ♀ with WT♂ zebrafish. *shank3*$^{+/-}$♂ were obtained from crosses of *shank3b*$^{-/-}$♂ with WT♀ zebrafish. There were no significant differences between the two groups in **(B'-D')** activity test and **(E'-F')** light/dark response test (except in the second light response). N=24 for each group.

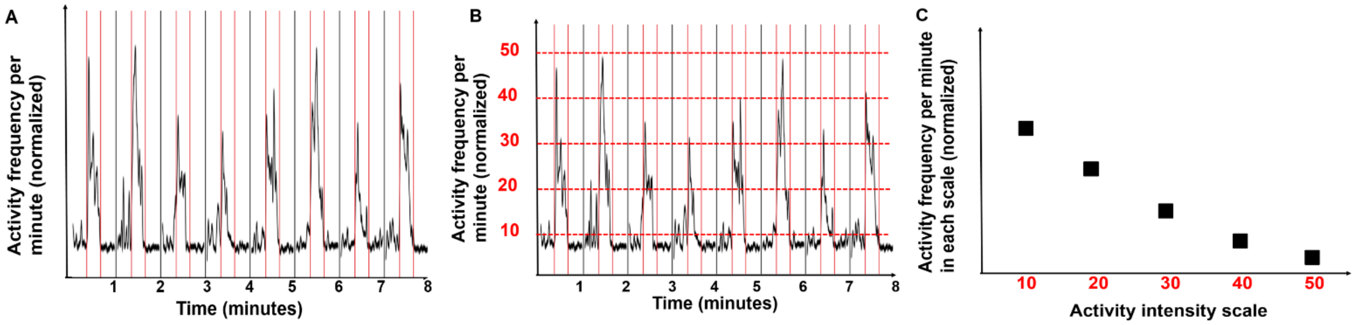

Additional file 1: Figure S6. Analysis of activity frequency at different activity intensity scale. Viewpoint's zebrabox was used to track the larvae and ViewPoint's zebralab system was used to analyze the larvae activity, as reported before [1, 2]. **(A)** The X-axis shows the time, and the Y-axis shows the activity frequency traveled by larvae in each 1-minute time bin. **(B)** To further analyze the variances of different activity intensity scales among WT, *shank3b*$^{+/-}$ and *shank3b*$^{-/-}$ zebrafish, we divided the activity equally into five levels (10, 20, 30, 40 and 50). **(C)** Next, we calculated the activity frequency of different activity intensity scales.

[1]. Rihel, J., et al., Zebrafish Behavioral Profiling Links Drugs to Biological Targets and Rest/Wake Regulation. Science, 2010.

[2]. Kokel, D., et al., Rapid behavior-based identification of neuroactive small molecules in the zebrafish. Nature Chemical Biology, 2010.
